# Endogenous hydrogen sulfide improves vascular remodeling through PPARδ/SOCS3 signaling

**DOI:** 10.1016/j.jare.2020.06.005

**Published:** 2020-06-20

**Authors:** Danyang Tian, Xu Teng, Sheng Jin, Yuhong Chen, Hongmei Xue, Lin Xiao, Yuming Wu

**Affiliations:** a Department of Physiology, Hebei Medical University, Shijiazhuang, China; b Intensive Care Unit, The Fourth Hospital of Hebei Medical University, Shijiazhuang, China; c Hebei Collaborative Innovation Center for Cardio-Cerebrovascular Disease, Shijiazhuang, China

**Keywords:** Hydrogen sulfide, Vascular remodeling, PPARδ, SOCS3, H_2_S, Hydrogen sulfide, CSE, Cystathionine-γ-lyase, PPG, DL-propargylglycine, VSMC, Vascular smooth muscle cell, MMP, Matrix metallopeptidase, ECM, Extracellular matrix, PPARδ, Peroxisome proliferator activated receptor delta, SOCS, Suppressor of cytokine signaling, STAT, Signal transducers and activators of transcription

## Abstract

**Introduction:**

Mounting evidences demonstrated the deficiency of hydrogen sulfide (H_2_S) facilitated the progression of cardiovascular diseases. However, the exact effects of H_2_S on vascular remodeling are not consistent.

**Objectives:**

This study aimed to investigate the beneficial role of endogenous H_2_S on vascular remodeling.

**Methods:**

CSE inhibitor, DL-propargylglycine (PPG) was used to treat mice and vascular smooth muscle cells (VSMCs). Sodium hydrosulfide (NaHS) was given to provide hydrogen sulfide. Vascular tension, H&E staining, masson trichrome staining, western blot and CCK8 were used to determine the vascular remodeling, expressions of inflammatory molecules and proliferation of VSMCs.

**Results:**

The deficiency of endogenous H_2_S generated vascular remodeling with aggravated active and passive contraction, thicken aortic walls, collagen deposition, increased phosphorylation of STAT3, decreased production of PPARδ and SOCS3 in aortas, which were reversed by NaHS. PPG inhibited expression of PPARδ and SOCS3, stimulated the phosphorylation of STAT3, increased inflammatory molecules production and proliferation rate of VSMCs which could all be corrected by NaHS supply. PPARδ agonist GW501516 offered protections similar to NaHS in PPG treated VSMCs. Aggravated active and passive contraction in PPG mice aortas, upregulated p-STAT3 and inflammatory molecules, downregulated SOCS3 and phenotype transformation in PPG treated VSMCs could be corrected by PPARδ agonist GW501516 treatment. On the contrary, PPARδ antagonist GSK0660 exhibited opposite effects on vascular contraction in aortas, expressions of p-STAT3 and SOCS3 in VSMCs compared with GW501516.

**Conclusion:**

In a word, endogenous H_2_S protected against vascular remodeling through preserving PPARδ/SOCS3 anti-inflammatory signaling pathway. Deficiency of endogenous H_2_S should be considered as a risk factor for VSMCs dysfunction

## Highlight

• H_2_S deficiency derived from CSE depletion contributes to the vascular remodeling and transformation of vascular smooth muscle cells.
• PPARδ/SOCS3 signaling pathway is decreased in CSE/H_2_S deficiency-associated transformation of VSMCs.
• Reduced PPARδ is responsible for decreased SOCS3 expression under condition of CSE depletion.
• CSE/H_2_S preserves SOCS3 production through PPARδ and inhibits inflammatory molecules production.

## Graphical abstract

**Figure:**
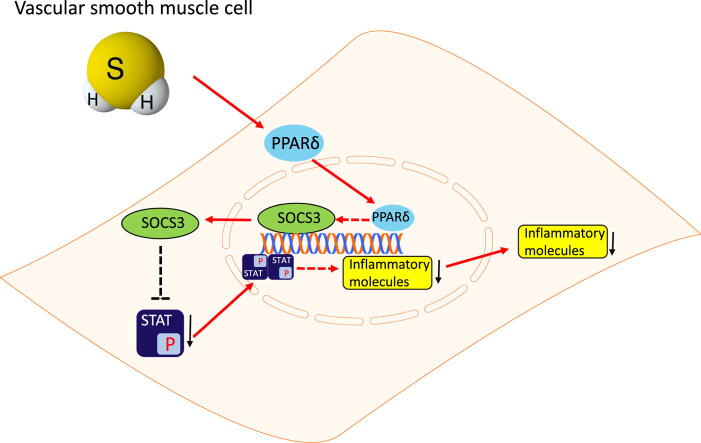

## Introduction

Excessive proliferation of vascular smooth muscle cells (VSMCs) and synthesis of extracellular matrix (ECM) promote progress of chronic diseases, like atherosclerosis, pulmonary hypertension, diabetes mellitus and so on [1], [2], [3]. According to the Poiseuille law, minor changes of blood vessel radius would lead to dramatic changes in resistance of blood flow, so contraction of VSMCs affects blood pressure as a principal factor. Recent studies suggested highly differentiated VSMCs could dedifferentiate into proliferating form and reenter the cell cycle in pathological conditions like artery injury or vascular remodeling models [4], [5], [6].

Peroxisome proliferator activated receptor delta (PPARδ) is a nuclear receptor playing important roles in glucose and lipid metabolism and it was found that PPARδ could increase the sensitivity of insulin, promote lipid metabolism and suppress inflammatory reaction [7]. More evidences indicate the protective roles of PPARδ in blood vessels of cardiovascular disease, and activating PPARδ with agonists or viral vector exerts effects of anti-inflammation, anti-atherosclerosis and anti-oxidation [8]. Inflammation attributes to transformation of VSMCs from contractile phenotype to synthetic phenotype via Ca^2+^ dependent or independent pathways, through which is vascular remodeling and contraction mechanism affected in progression of hypertension [9], [10], [11].

Suppressor of cytokine signaling 3 (SOCS3) belongs to SOCS family which consists of 8 proteins suppressing Janus kinase/signal transducer and activator of transcription (Jak/STAT) signaling pathway [12]. Numerous studies demonstrate the anti-inflammatory effect of SOCS3 in hypertension, obesity and allergic reaction bringing more insights into the role of suppressing inflammation [13]. SOCS3 preserves endothelial function in angiotensin II (Ang II) induced hypertension model, suppresses inflammation of macrophages in lipopolysaccharides (LPS) induced model and inhibits proliferation of VSMCs in vein graft restenosis model [14], [15], [16].

Hydrogen sulfide (H_2_S), as the third labeled endogenous gasotransmitter, performs numerous physiological activities in cardiovascular and other systems. The favorable roles of H_2_S on cardiovascular system are associated with anti-inflammation, anti-oxidation, anti-apoptosis properties and regulating ion channel to defend ischemia-reperfusion injury, to improve prognosis of coronary heart disease and to delay progress of atherosclerosis [17], [18]. H_2_S is generated by three enzymes: cystathionine-β-synthase (CBS), 3-mercaptopyruvate sulfurtransferase (MPST) and cystathionine-γ-lyase (CSE). CSE is mainly responsible for H_2_S production in cardiovascular system. Global knockout of CSE caused elevation of systolic blood pressure, high level of oxidative stress and inflammation, accompanying with low level of H_2_S in plasma. On the contrary, administration of H_2_S causes protective effects against hypertension, heart failure and atherosclerosis [19].

Researchers find H_2_S inhibits deposition of calcium in ECM, suppresses osteoblast trans-differentiation and calcification of VSMCs [20], regulates K_ATP_ channels to relax vascular smooth muscles and reduces expression of mitogen-activated protein kinase (MAPK) to inhibit proliferation of VSMCs [21], [22]. H_2_S inhibits phosphorylation of STAT3 and expression of Jak to improve LPS-induced disorders of iron metabolism [23]. Administration of H_2_S donor GYY4137 could attenuate proliferation of hepatocellular carcinoma and restrict growth of tumor through inhibiting phosphorylation of STAT3 [24], [25]. Our previous study suggests beneficial roles of PPARδ on blood vessels. PPARδ could increase endothelial nitric oxide synthase (eNOS) production by activating phosphoinositide 3-kinase/protein kinase B (PI3K/Akt) or 5′ adenosine monophosphate-activated protein kinase (AMPK) signaling pathway in renal arterial endothelium of spontaneous hypertension rats [26]. Although the beneficial effects of H_2_S have been investigated in many types of cells, however the exact mechanisms of anti-inflammatory effect on VSMCs are not clear. This study aims to explore the involvement of PPARδ and SOCS3 in mediating the anti-inflammatory effect of endogenous H_2_S in improving vascular remodeling.

## Materials and methods

### Animals and treatment

C57BL/6J mice were purchased from Vitalriver Company (Beijing, China). All animals were housed under standard conditions of room temperature 20 ± 8 °C, humidity 60 ± 10%, lights with a 12 h light/12 h dark cycle and free access to diet and water. All animal procedures are followed the Animal Management Rule of the Ministry of Health, People’s Republic of China (documentation number 55, 2001) and the Care and Use of Laboratory Animals approved by the Animal Care Committee of Hebei Medical University. The investigation conforms NIH Guide for the Care and Use of Laboratory Animals.

At the age of 12 weeks, C57BL/6J mice were randomly assigned to 4 groups: Vehicle group, NaHS group (56 μmol/kg/day), PPG group (20 mg/kg/day), PPG + NaHS group. DL-propargylglycine (PPG) was given by intraperitoneal injection and the treatment lasted for 8 weeks from the age of 12 weeks. Sodium hydrosulfide was given by intraperitoneal injection and the treatment lasted for 4 weeks from the age of 16 weeks. This dose of NaHS and PPG are safe concerning in vivo animal treatment as referenced to similar studies.

### Blood pressure measurement

Blood pressure was measured by the tail-cuff end plethysmography (BP-100A; Taimeng Software, Chengdu, China). Mice were kept in quiescent conditions. The average of 3 records was taken as the systolic blood pressure, diastolic blood pressure and mean blood pressure of each mice.

### Mice aorta preparation and measurement of vascular contraction and remodeling

Animals were sacrificed by CO_2_ suffocation at the age of 20 weeks. Thoracic aortas were removed of surrounding connective tissues and dissected in oxygenated ice-cold Krebs solution filled with (mM): 119 NaCl, 4.7 KCl, 2.5 CaCl_2_, 1 MgCl_2_, 25 NaHCO_3_, 1.2 KH_2_PO_4_, and 11 D-glucose. Briefly, each artery was cut into segments with the length of about 1.6 mm after the surrounding connective tissues removed. Then the rings were suspended with two stainless steel wires inserting through the artery lumen and fixing to jaws of the myograph in the Multi Myograph System (610 M, Danish Myo Technology A/S, Aarhus N, Denmark) for isometric force measurement. The organ chamber was filled with 5 mL of Krebs solution, gassed with 95% O_2_ and 5% CO_2_ at 37 °C (pH ~ 7.4). The rings were undergone 3 mN stretch and 60 min-equilibration before each experiment.

After contraction of 60 mM KCl to a steady state, fresh Krebs solution were used to wash for 3 times. Concentration-dependent contractions evoked by accumulative phenylephrine (Phe, 0.001–10 μM) were recorded and vascular active contraction was expressed as percentage increase in 60 mM KCl-generated contraction. The relaxation percentage of concentration-gradient sodium nitroprusside (0.001–1 μM) was also calculated after Phe contraction. Gradually stretched (10 μm every 10 min) for 15 times to increase the internal circumference of segment and vascular passive contraction was calculated as force to 60 mM KCl-generated contraction.

### Organ culture of aortic rings

Mice aortic rings (1.6 mm in length) were dissected in sterile phosphate buffered saline (PBS) and incubated in low-glucose Dulbecco’s Modified Eagle’s Media (DMEM, Gibco, Gaithersberg, MD, USA) supplemented with 10% fetal bovine serum (FBS, Gibco) and 100 IU/mL penicillin plus 100 μg/ml streptomycin. Aortic rings were treated with PPG (1 mM), NaHS (50 μM), GW501516 (1 μM, MedChemExpress, Shanghai, China) and GSK0660 (1 μM, MedChemExpress, Shanghai, China).

### Cell culture and passage

VSMCs were isolated from aortas of 100–120 g male Sprague-Dawley (SD) rats sacrificed by CO_2_ suffocation. Thoracic aortas were immediately removed of surrounding connective tissues and dissected in sterile PBS. VSMCs were cultured in DMEM (Gibco) supplemented with 10% FBS (Gibco) and 100 IU/mL penicillin plus 100 μg/mL streptomycin in incubator (Thermo 321, USA) with a humidified atmosphere of 5% CO_2_ at 37 °C. Cells at passages 5–8 with 40–60% confluence were used in the experiment.

### Measurement of H_2_S from mice

Whole blood sample was collected, anticoagulated, and centrifuged with Heraeus Pico 17 (Thermo Scientific, USA) at 3000 rpm for 15 min to collect plasma. H_2_S levels in plasma were measured by HPLC using Ultimate 3000 (Thermo Scientific, USA) liquid chromatography system with pre-column derivatization as Tan et al. reported [27]. The acquisition of chromatographic data was performed by means of Chromeleon software (ver.7.0, Thermo Electron). H_2_S levels were calculated using a standard curve generated from a sodium sulfide solution (0–100 μmol/L)

### Morphologic examination

The mice thoracic aortas were imbedded with Tissue Freezing Medium (SAKURA, USA) for frozen sections (10 μm thick, LEICA CM 1950, Germany) and fixation was prepared in 4% paraformaldehyde, washed with PBS. Then hematoxylin and eosin (H&E) and masson trichrome staining were used to measure wall thickness and collagen deposition of aorta.

### VSMCs proliferation assay

Cell counting kit-8 kits (CCK-8, MedChemExpress, Shanghai, China) were used to evaluate VSMCs proliferation. VSMCs were plated onto 96-well cell culture plate at a density of 2 × 10^3^ cells/well for 24 h at 37 °C, and treated with different concentrations of chemicals for 24 h. After washing with PBS, a total volume of 100 μL DMEM containing 10 μL of CCK-8 solution was added to each well, and incubated for 2 h. Finally, the absorbance was conducted at wavelength of 450 nm with spectrophotometer (Bio-Rad, USA).

### Western blot analysis

Isolated mouse aortas, or VSMCs were homogenized in RIPA lysis buffer that contained 1 μg/mL leupeptin, 5 μg/mL aprotinin, 100 μg/mL PMSF, 1 mM sodium orthovanadate, 1 mM EDTA, 1 mM EGTA, 1 mM sodium fluoride, and 2 μg/mL β-glycerolphosphate. After homogenates centrifuged at 20,000 g for 20 min at 4 °C, supernatant was harvested and kept at −80 °C lab refrigerator (Haier DW-86L959W, Qingdao, China). Protein concentration was determined by the bicinchoninic acid method (Generay Biotechnology, Shanghai, China). Equal amount of protein samples was electrophoresed on a 10% SDS-polyacrylamide gel and transferred onto PVDF membrane (Millipore, Billerica, Massachusetts, USA) by wet transfer at 110 V for 90 min at 4 °C. Blots were blocked with 1% bovine serum albumin for 1 h and incubated overnight at 4 °C with antibodies against CSE (1:1000; Proteintech, Wuhan, China), CBS (1:1000; Proteintech), MPST (1:1000; Proteintech), Collagen I (1:1000; Proteintech), matrix metallopeptidase (MMP9, 1:1000; Proteintech), α-Smooth muscle actin (αSMA, 1:2000; Proteintech), p27 (1:2000; Proteintech), proliferating cell nuclear antigen (PCNA, 1:1000; Proteintech), Cyclin E (1:1000; Proteintech), tumor necrosis factor α (TNFα, 1:2000; Proteintech), interleukin 6 (IL6, 1:1000; Proteintech), IL1β (1:500; Proteintech), PPARδ (1:1000; Proteintech), SOCS3 (1:1000; Proteintech), glyceraldehyde-3-phosphate dehydrogenase (GAPDH, 1:3000; Proteintech), and phosphorylated STAT3 (p-STAT3, 1:2000; Wanleibio, Shenyang, China). After washing with TBST 3 times, blots were incubated with HRP-conjugated anti-rabbit, anti-mouse, or anti-goat IgG (1:3000, Proteintech) for 1 h at room temperature. Immunoreactive bands were visualized by chemiluminescence (ECL reagents, WBKLS0500, Millipore, Massachusetts, USA) and exposed to an X-ray film for densitometric analysis.

## Statistical analysis

All data are presented as means ± SEM. Statistical analysis was performed by Student *t*-test and one-way ANOVA, followed by Bonferroni post hoc tests when more than two treatments were compared (GraphPad Software, San Diege, CA). *P* < 0.05 was considered statistically significant.

## Results

### Deficiency of endogenous hydrogen sulfide induced elevation of blood pressure and vascular contraction

**Fig. 1:**
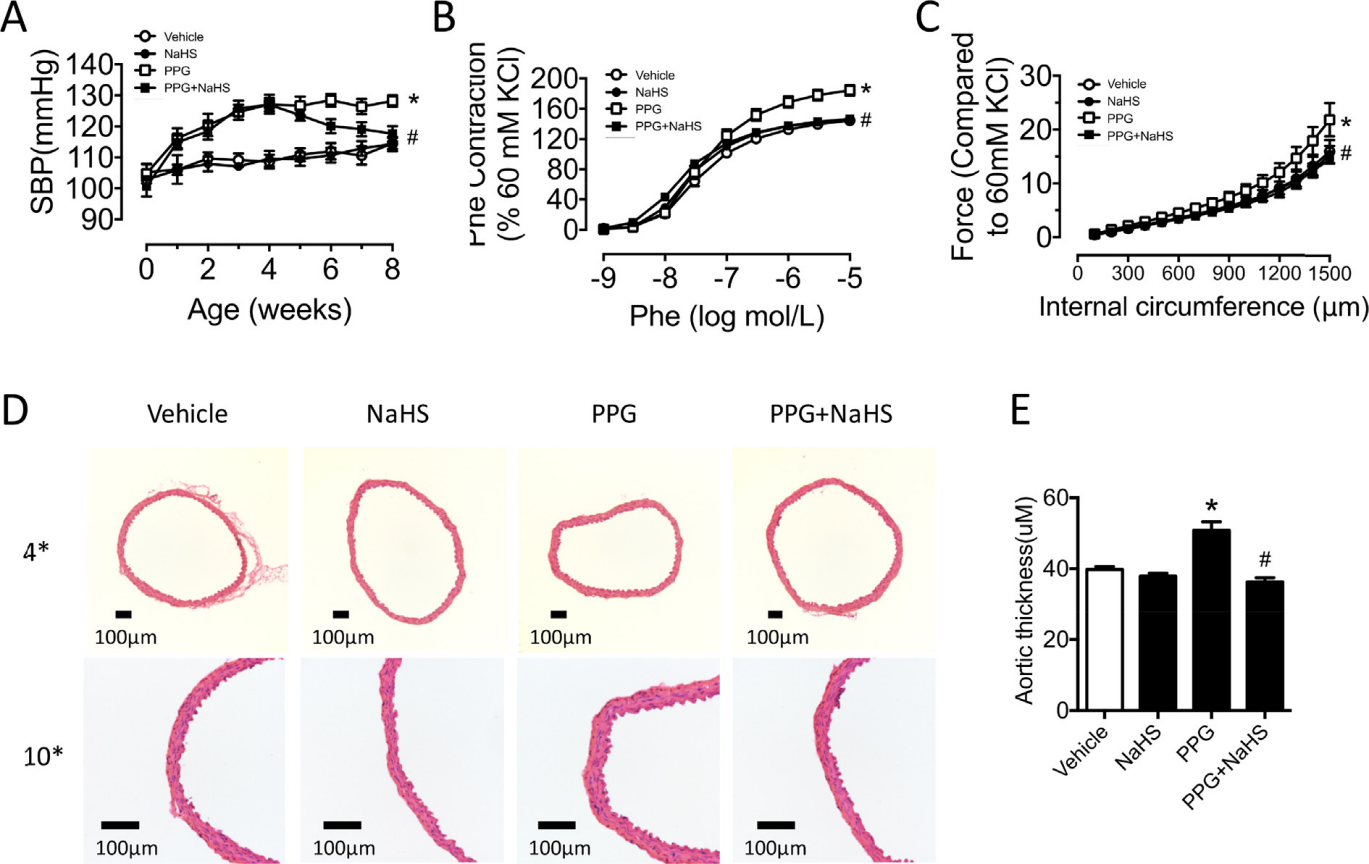
Deficiency of endogenous hydrogen sulfide induced elevation of blood pressure and vascular contraction. A: PPG group mice exhibited higher blood pressure which were modified by NaHS chronic treatment. B: Phe-induced contraction in PPG mice aortas demonstrated enhanced active contraction which were corrected by NaHS administration. C: aggravated stretch-induced passive contraction in PPG aortas were reversed by NaHS supply. D: representative images of H&E staining of aortic sections. E: analysed result indicated thicken aortic walls in the PPG mice aortas. Data are means ± SEM. n = 8 for vascular contraction in each group and n = 6 for other groups. *P < 0.05 versus vehicle, ^#^*P* < 0.05 versus PPG.

To investigate the function of endogenous hydrogen sulfide on vascular smooth muscle cells, specific CSE inhibitor PPG was in vivo used via intraperitoneal injection on C57BL/6J mice. In vivo PPG treatment (20 mg/kg) for 4 weeks induced obvious decreased expression of CSE in the aortas, whereas no changes of the other two hydrogen sulfide synthases CBS and MPST were found (Fig. S1A–C). What is more, PPG group were validated with decreased plasma hydrogen sulfide by HPLC (Fig. S1D). In addition, elevated blood pressure was presented in PPG treated mice, which could be reversed by NaHS ex vivo injection (Fig. 1A and Fig. S1E). Vascular tension when administrated with accumulative Phe was recorded to represent vascular active contraction in different conditions. Strengthened contractile ability was observed in PPG group which was corrected by NaHS complement (Fig. 1B). Stretch assay demonstrated that enhanced vascular passive contraction was detected with PPG treatment which could be normalized by NaHS supplement (Fig. 1C). The majority of vascular wall was composed with smooth muscle cells. PPG group exhibited thicken vascular wall with H&E staining, which were reversed by NaHS treatment (Fig. 1D-E). However, no significant changes were observed in the ratio of lumen/outer diameter of all groups (Fig. S1G).

### Deficiency of hydrogen sulfide induced vascular remodeling

**Fig. 2:**
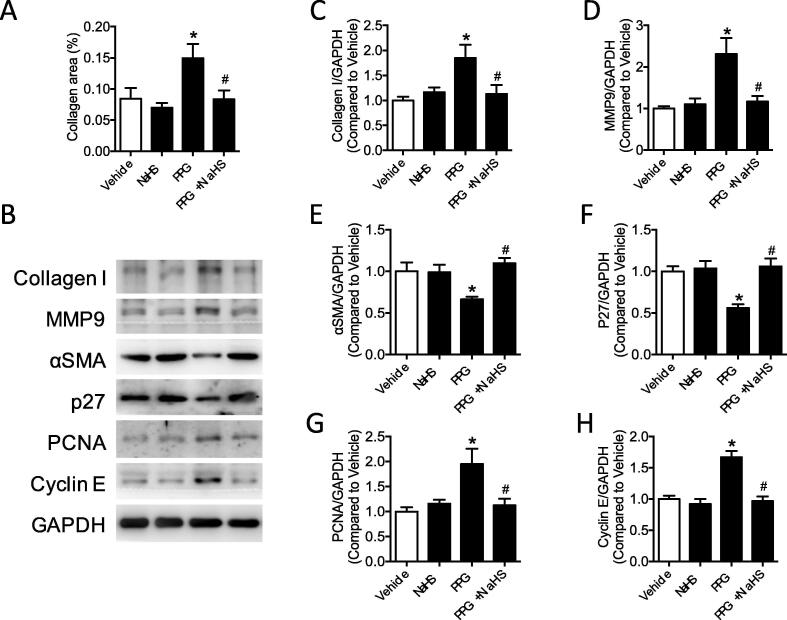
Deficiency of hydrogen sulfide induced vascular remodeling. A: collagen areas (ratio to the vessel area) of aortic sections were increased in PPG group. B: western blot bands of collagen I, MMP9, αSMA, p27, PCNA, Cyclin E in aortas. C-H: expressions of collagen I, MMP9, PCNA, Cyclin E were increased, while the production of αSMA, p27 were decreased in PPG treated aortas. Data are means ± SEM. n = 6 for each group. *P < 0.05 versus vehicle, ^#^*P* < 0.05 versus PPG.

Collagen composition in the aortic section was determined with masson trichrome staining demonstrating that overproduced collagen in the PPG mice could be normalized to the control level after NaHS treatment (Fig. 2A). Western blot band also validated the increased collagen I production in the PPG mice aortas compared with vehicle mice aortas (Fig. 2C). Besides, MMP 9 represented the same trendency with collagen I (Fig. 2D). As the important markers of vascular remodeling, vascular smooth muscle cells phenotype proteins in aortas were detected by western blot, stating decreased αSMA, p27 and increased PCNA, Cyclin E in the PPG group which could be corrected by NaHS treatment (Fig. 2E–H).

### PPG promoted inflammatory molecules production and inhibited PPARδ, SOCS3 expression of aortas

**Fig. 3:**
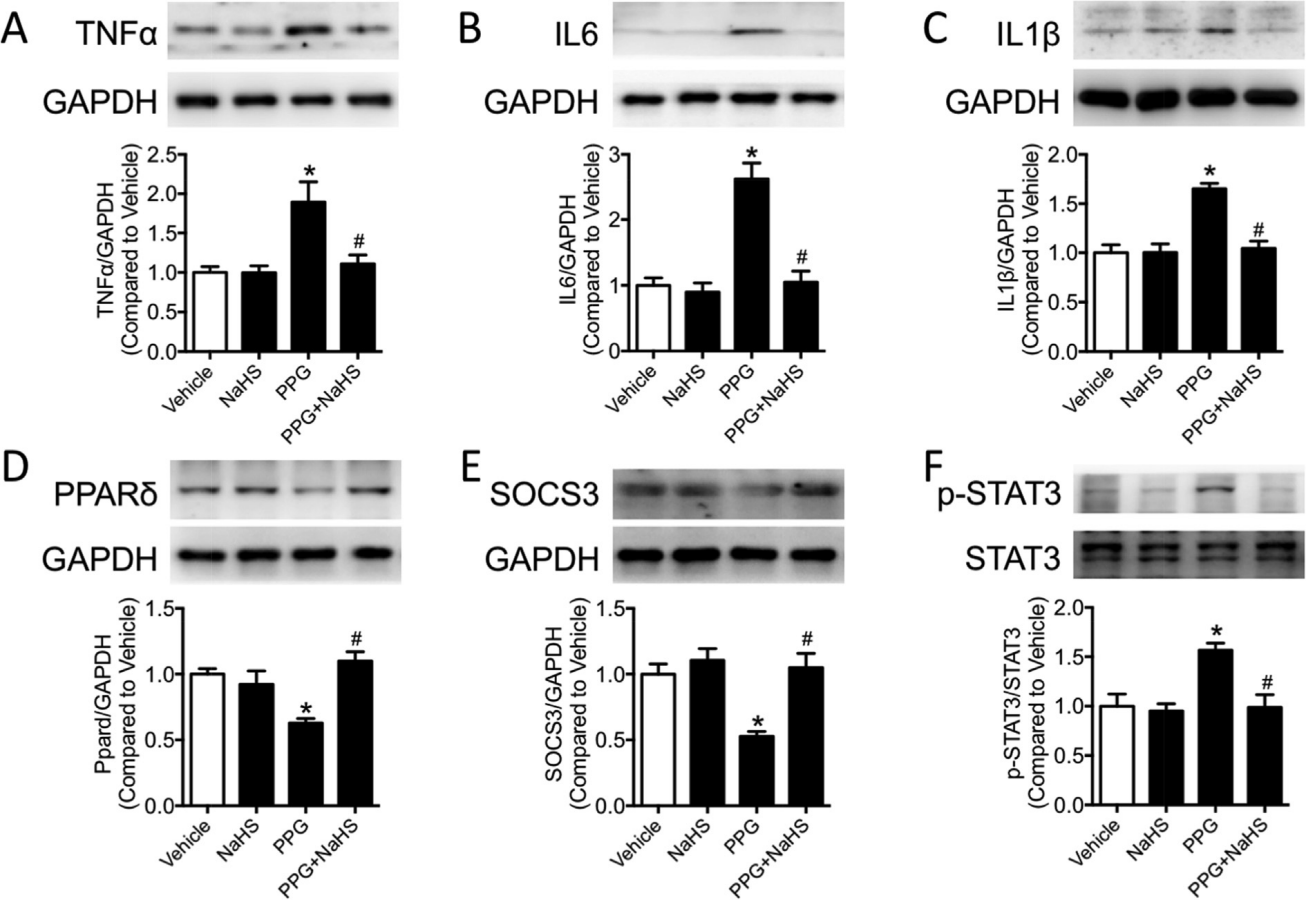
PPG promoted inflammatory molecules production and inhibited PPARδ, SOCS3 expression of aortas. A-F: western blot bands and quantification showed increased TNFα, IL6, IL1β and phosphorylation of STAT3 (Ser 727) and decreased PPARδ, SOCS3 in PPG treated aortas. Data are means ± SEM. n = 6 for each group. *P < 0.05 versus vehicle, ^#^*P* < 0.05 versus PPG.

Mounting evidences suggested inflammatory molecules promoted vascular remodeling in pathological state. To investigate whether inflammatory molecules are involved in the vascular remodeling progress in the PPG mice aortas, inflammatory molecules TNFα, IL6 and IL1β were examined by western blot. Western blot results showed that inflammatory molecules TNFα, IL6 and IL1β were raised in PPG mice, which were recovered in PPG + NaHS mice (Fig. 3A–C). STAT3, a crucial promoter for inflammatory molecules, was detected with increased phosphorylation in PPG group demonstrating aggravated inflammation existed in aortas lack of hydrogen sulfide (Fig. 3F). SOCS3, an inhibitor of STAT3 which finally prevented inflammatory molecules transcription in many metabolic diseases, were found decreased after PPG injection and could be reversed by NaHS treatment (Fig. 3E). PPARδ, as a nuclear receptor playing important role in transcription, represented the same tendency with SOCS3. It was suppressed in PPG treatment group but was corrected after NaHS supply (Fig. 3D).

### PPARδ attenuated vascular contraction in PPG treated aortas

**Fig. 4:**
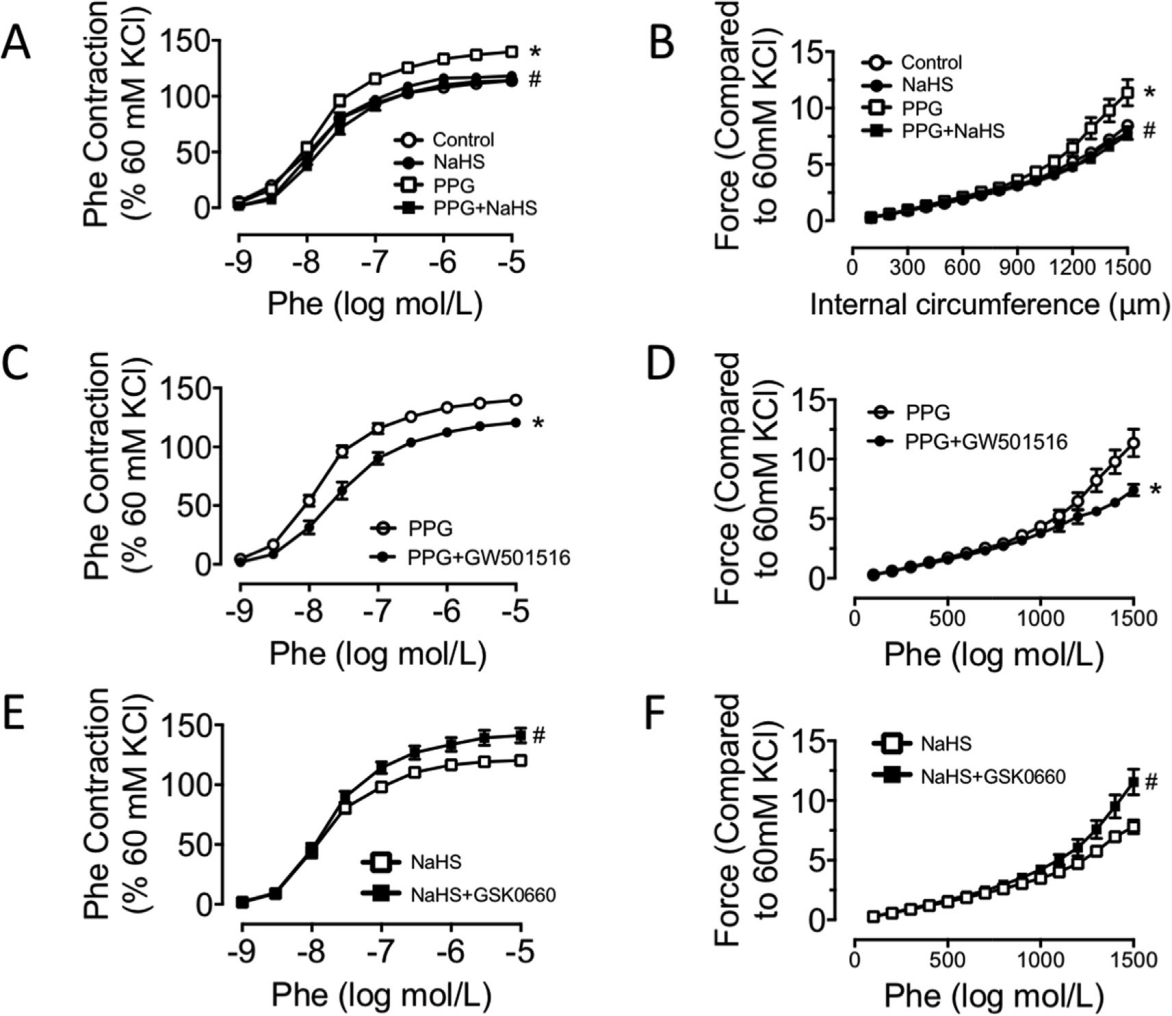
PPARδ attenuated vascular contraction in PPG treated aortas. A: enhanced Phe-induced contraction of aortas in PPG group were corrected by NaHS supply. B: aggravated stretch assay of aortas in PPG group were corrected by NaHS supply. C-D: GW501516 improved Phe-induced contraction and passive contraction of aortas in PPG mice. E-F: Phe-induced contraction and passive contraction of aortas in NaHS mice were aggravated by GSK0660 stimulation. Data are means ± SEM. n = 8 for each group. A-B: *P < 0.05 versus control, ^#^*P* < 0.05 versus PPG. C-D: *P < 0.05 versus PPG. E-F: ^#^*P* < 0.05 versus NaHS.

To make it clear whether PPARδ serves as protective site in vascular contraction, PPARδ agonists and antagonists were used. There was an obvious elevation of active contraction administrated with accumulated dose of Phe and a significant increase of passive contraction in stretch assay in the ex vivo PPG culture for 24 h. No changes were observed in the NaHS culture group (Fig. 4A–B). GW501516 is a well-characterized and highly specific PPARδ activator. Culture with GW501516 (1 μM) for 24 h in aortas of PPG treated mice caused an obvious reduction of both active and passive contraction (Fig. 4C–D). In contrast, culture with PPARδ antagonist GSK0660 (1 μM) 24 h in aortas of NaHS treated mice caused an obvious elevation of both active and passive contraction (Fig. 4E-F).

### PPG induced collagen production and phenotype transformation of VSMCs

**Fig. 5:**
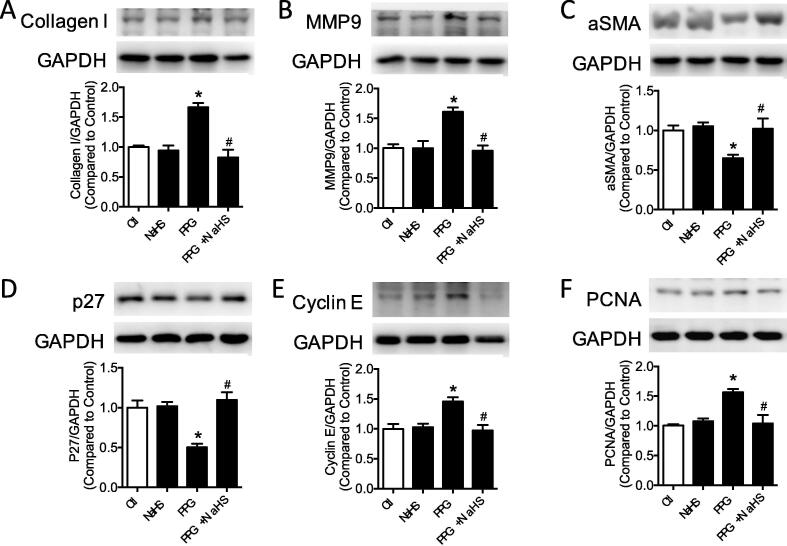
PPG induced collagen production and phenotype transformation of VSMCs. A-F: expression of collagen I, MMP9, PCNA, Cyclin E were increased, while the production of αSMA, p27 were decreased in PPG stimulated VSMCs. Data are means ± SEM. n = 6 for each group. *P < 0.05 versus control, ^#^*P* < 0.05 versus PPG.

PPG were administrated to explore the effect of hydrogen sulfide deficiency on collagen production and phenotype transformation in the vascular smooth muscle cells (VSMCs). Time dependent and dose dependent assay confirmed the shortest period and lowest dose of PPG administration to induce the most obvious changes, which were administrated in the latter experiment (Fig. S2A–F). As the irreversible CSE inhibitor, the effect of PPG was validated by western blot result of CSE, CBS and MPST (Fig. S3A–D). Ex vivo PPG treatment (1 mM) for 24 h stimulated collagen I and MMP9 expression, accompanying with elevation of proliferation markers PCNA and Cyclin E (Fig. 5A–B, E-F, S4B-C, S4F-G). On the contrary, contraction markers αSMA and p27 were reduced in PPG treatment which could be reversed by NaHS supply (Figs. 5C–D, S4D–E).

### PPG promoted inflammatory molecules production and inhibited PPARδ, SOCS3 expression of VSMCs

**Fig. 6:**
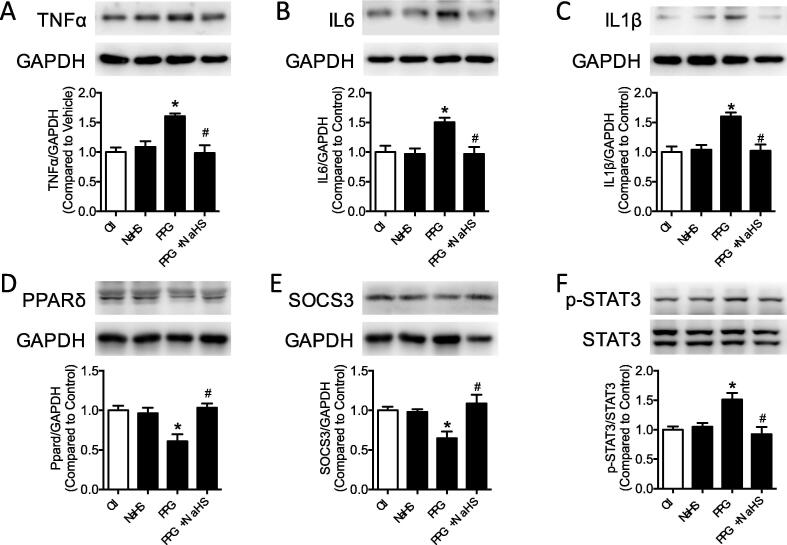
PPG promoted inflammatory molecules production and inhibited PPARδ, SOCS3 expression of VSMCs. A-F: increased TNFα, IL6, IL1β and phosphorylation of STAT3 (Ser 727) and decreased PPARδ, SOCS3 in PPG treated VSMCs were reversed by NaHS supply. Data are means ± SEM. n = 6 for each group. *P < 0.05 versus control, ^#^*P* < 0.05 versus PPG.

Consisted with production of inflammatory molecules TNFα, IL6 and IL1β in PPG stimulated aortas, hydrogen sulfide deficiency caused inflammation in VSMCs (Figs. 6A–C, S5A–D). NaHS supplement could normalize expression of inflammatory molecules. Compared with control group, PPARδ and SOCS3 were decreased in PPG group which could be reversed to the normal level after NaHS supply (Figs. 6D–E, S5E–F). Overexpressed phosphorylation of STAT3 were detected in PPG treatment (Fig. 6F, S5G). PPARδ, SOCS3 and p-STAT3 in the NaHS group presented no obvious changes compared with control group.

### PPARδ attenuated inflammation and proliferation in VSMCs

**Fig. 7:**
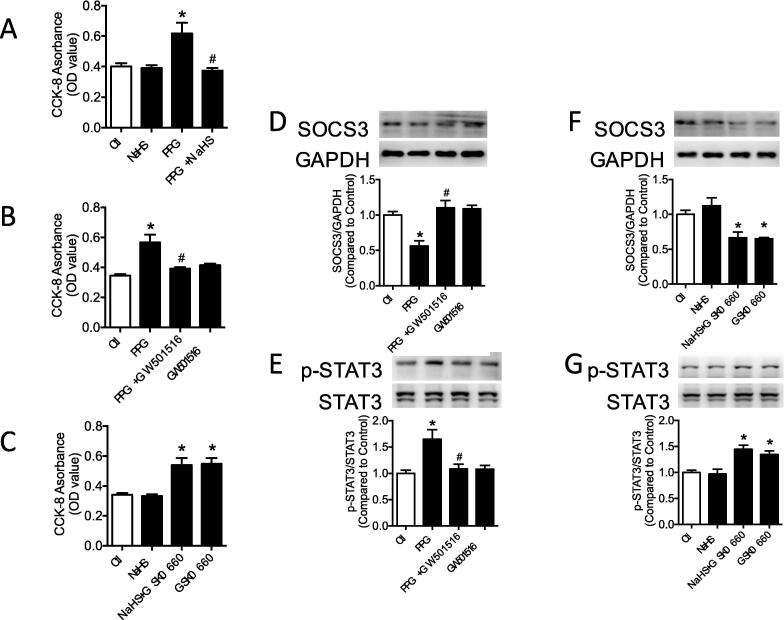
PPARδ attenuated inflammation and proliferation in VSMCs. A-C: elevated proliferation of VSMCs in PPG group were normalized by GW501516. Proliferation of VSMCs were aggravated by GSK0660 culture in NaHS group. D-E: decreased expression of SOCS3 and increased phosphorylation of STAT3 in PPG culture could be corrected by GW501516 in VSMCs. F-G: GSK0660 stimulation decreased SOCS3 expression and increased p-STAT3 expression in NaHS and control group. Data are means ± SEM. n = 9 for proliferation rate and n = 6 for other groups. *P < 0.05 versus control, ^#^*P* < 0.05 versus PPG.

VSMCs proliferation were valiadated by CCK-8 assay. As showed in Fig. 7A, VSMCs in PPG group presented higher proliferation rate compared with control group and enhanced proliferation rate in PPG group could be normalized by NaHS administration. To make it clear whether PPARδ serves as upstream of SOCS3, PPARδ agonist and antagonist were used to incubate VSMCs and we measured proliferation rate, expression of SOCS3, p-STAT3. PPARδ agonist, GW501516 markedly attenuated VSMCs proliferating rate, inhibited phosphorylation of STAT3 and recovered SOCS3 expression in PPG group (Fig. 7B, D–E). PPARδ antagonist, GSK0660, stimulated VSMCs proliferation and phosphorylation of STAT3 significantly and downregulated SOCS3 expression in NaHS group (Fig. 7C, F–G). However, single usage of GW501516 or NaHS caused no obvious changes of VSMCs proliferation and expression of SOCS3 and p-STAT3.

### PPARδ attenuated inflammatory molecules production, collagen production and phenotype transformation of VSMCs

The increased collagen I, MMP9, PCNA and Cyclin E in PPG treated VSMCs could be totally reversed with the presence of GW501516. While the declined αSMA and p27 in PPG treated VSMCs could be corrected by GW501516 (Fig. S6A–F). On the contrary, collagen I, MMP9, PCNA and Cyclin E were enhanced by GSK0660 in the presence of NaHS. αSMA and p27 were downregulated by GSK0660 in the presence of NaHS (Fig. S8A–F). The increased inflammatory molecules TNFα, IL6 and IL1β in PPG treated VSMCs were normalized by GW501516 culture (Fig. S7A–C). And the inflammatory molecules TNFα, IL6 and IL1β in NaHS group were elevated with GSK0660 stimulation (Fig. S9A–C). The expression of hydrogen sulfide synthase CSE was only affected by PPG, but not GW501516 or GSK0660. The other two synthases CBS and MPST were not affected by PPG, GW501516 or GSK0660 (Fig. S7D–F, S9D–F).

## Discussion

VSMCs are the major cells locating in the media layer to form vascular walls and the aggravation of VSMCs contraction is crucial for hypertension development and progression [28]. VSMCs are sensitive to many factors: renin-Ang II-aldosterone, sympathetic nervous system, oxidative stress, inflammatory molecules and hemodynamics, and are prone to phenotype transformation in pathological conditions [11], [29]. In our study, elevation of systolic blood pressure and vascular contraction were observed in H_2_S deficiency mice. Aortas in PPG mice presented enhanced Phe-induced contraction and stretch-induced contraction which represent the active tension and passive tension of vessels respectively. The dysfunction of vascular contraction is associated with vascular remodeling consisting of phenotype transformation from contractile to synthetic and ECM deposition. Contractile VSMCs are responsible for maintaining vasomotor tone and blood pressure, while synthetic VSMCs take part in the progress of proliferation and migration [30]. Mature VSMCs present contractile proteins such as: αSMA, p27, SM22α and smooth muscle myosin heavy chain (SMMHC) and could dedifferentiate to synthetic phenotype characterized by PCNA, Cyclin E, Cyclin D1 and OPN in pathological conditions [31]. In our study, synthetic genes PCNA and Cyclin E were overproduced and contractile genes αSMA and p27 were decreased in PPG treated VSMCs. Ex vivo administration of NaHS could reversed the phenotype transformation of VSMCs deficient in H_2_S. CCK8 is an extensively applied kit which reveals NaHS supply could normalize the exacerbating proliferation of PPG-induced VSMCs and this assay proved that H_2_S deficiency triggered VSMCs phenotype transformation and proliferation.

ECM, especially collagen deposition influences occurrence of hypertension to a great extent via promoting passive tension of aorta and attenuating elasticity of blood vessels [32]. MMP9, named matrix metallopeptidase 9, belongs to a class of enzymes which are involved in the degradation of the extracellular matrix and inhibition of collagen deposition. However, MMP9, in accordance with collagen I, were elevated in transverse aortic constriction and hypertensive vascular remodeling [33], [34]. Elevated MMP9 participates in the composition of collagen and degradation of elastin which induces abnormal arterial compliance and aggravated fibrosis [35]. Collagen deposition were observed in PPG mice aortas stained by masson trichrome, which were validated by western blot demonstrating elevated collagen I and MMP9 in PPG group. It was proven that endogenous H_2_S deficiency caused collagen deposition in aorta which further elevated blood pressure.

Inflammation is the initial cause of vascular remodeling. TNFα, IL6 and IL1β were elevated in PPG mice aortas which could be reversed by NaHS supply indicating that deficiency of H_2_S was the cause of inflammation. Inflammatory molecules could activate various cellular pathways to induce expression of vasoconstrictors like endothelin and Ang II in the endothelium; to activate Ca^2+^ channel, protein kinase C (PKC), Rho kinase and MAPK pathway promoting cellular proliferation and migration; to interact with integrin through MMPs to influence ECM deposition [30], [36]. Increased inflammatory molecules are associated with vascular dysfunction and remodeling in atherosclerosis, hypertension and abdominal aneurysm [37]. TNFα is a cellular signaling factor mediating inflammation during proliferation, differentiation and apoptosis [38]. IL6 is a crucial pro-inflammatory factor contributing to host defense through the stimulation of acute phase response, immune reactions and hematopoiesis [39]. As a member of interleukin 1 family, IL1β is activated by NLR family pyrin domain containing 3 (NLRP3) inflammasome which leads to cleavage of pro-caspase 1 to its active form, finally promoting pain and fever through activating cyclooxygenase-2 (COX-2) [40]. TNFα could induce IL6 and IL1β as a cellular signaling molecule attributing to the inflammatory regulation in atherosclerosis [41], or act as a pro-inflammatory molecule to promote hypertension or atherosclerosis with IL6 and IL1β [42]. In our study, overproduced collagen and proteins of synthetic phenotype in PPG group illustrated that VSMCs phenotype transformation and vascular remodeling were induced by inflammatory molecules.

SOCS3, belonging to suppressor of cytokine signaling family, suppresses inflammation by inhibiting Jak/STAT signaling pathway. As a vital transcription factor of inflammation, STAT3 is activated by phosphokinase and undergo a conformational change to bind to another phosphorylated STAT3, then the dimer would be transferred from cytoplasm to nucleus and bind to promoter region of inflammatory molecules to induce transcription [12]. On one side SOCS3 inhibits activity of Jak directly, on the other side SOCS3 degrades Jak by ubiquitination to suppress downstream signaling [13]. PPARδ is a member of peroxisome proliferator activated receptor family, acting as a nuclear receptor regulating glucose and lipid metabolism [43]. PPARδ inhibits VSMCs proliferation through suppressing cyclin D1 and cyclin-dependent kinase 4, and inducing cyclin-dependent kinase inhibitor p21 and p27 [44]. PPARδ contributes to anti-inflammation and anti-apoptosis effects and suppresses cellular migration in metabolic diseases by inhibiting IL1β [45]. In diabetic mice, the phosphorylation of STAT3 is suppressed by PPARδ or its agonist GW501516 which blocks inflammation in adipocytes and hepatocytes [46].

Recent studies show H_2_S plays a protective function in renal arterial endothelium by activating the PPARδ/PI3K/Akt/eNOS or PPARδ/AMPK/eNOS pathway [26]. In our experiment, elevation of p-STAT3 induced by PPG could be reversed by NaHS administration. Compared with p-STAT3, SOCS3 and PPARδ represented the opposite changes that decreased expressions were found in PPG group which could be corrected by NaHS supply. The results indicated that deficiency of H_2_S activated inflammatory signaling through suppressing production of SOCS3 and PPARδ and elevating phosphorylation of STAT3. To study the roles of PPARδ played on the SOCS3 and STAT3, GW501516 and GSK0660 were used and the expressions of SOCS3 and p-STAT3 were detected. GW501516 increased the expression of SOCS3, suppressed phosphorylation of STAT3 and relieved contraction of PPG pretreated aortas. Conversely GSK led to decreased production of SOCS3, elevated production of p-STAT3 and aggravated vascular contraction in both NaHS pretreated and control aortas. Our results demonstrated endogenous H_2_S lessened vascular contraction and vascular remodeling through activating PPARδ/SOCS3 pathway to suppress inflammation.

The single usage of GW501516 didn’t affect expressions of SOCS3, p-STAT3 and changes of vascular contraction, indicating that PPARδ was highly expressed in VSMCs to inhibit phosphorylation of STAT3 thus suppressing downstream inflammatory molecules transcription through activating SOCS3 in physiology state. Expression of PPARδ would only be inhibited in the pathological case to activate inflammatory signaling further. In our experiment, the single usage of NaHS didn’t cause significant changes which were in accordance with the previous studies [47], [48]. We speculated that the main function of physiological dose of H_2_S was to maintain homeostasis of body. That explained why physiological dose of H_2_S administration caused no obvious changes of cells and only the deficiency of H_2_S brought abnormal changes or elevated susceptibility to inflammation [49].

The novelty is we firstly demonstrated that the endogenous H_2_S improves vascular remodeling and inhibits inflammatory reactions through activating PPARδ/SOCS3 signaling pathway. However, the limitation of our study is that it is still unknown how endogenous H_2_S regulates PPARδ, directly or through other signaling pathway. And future studies should be conducted to prove the protective role of H_2_S on arteriole.

## Conclusion

Deficiency of H_2_S led to decreased PPARδ and SOCS3 which caused increased phosphorylation of STAT3 and overproduced inflammatory molecules in VSMCs, thus leading to vascular remodeling and enhanced vascular contraction. Administration of PPARδ agonist GW501516 could activated SOCS3, suppressed p-STAT3 in PPG stimulated VSMCs and lessened vascular contraction in PPG treated aorta. In a word, endogenous H_2_S protected blood vessels from vascular remodeling through PPARδ/SOCS3 signaling pathway to exert an anti-inflammatory effect. This study provided new insights into the beneficial role of H_2_S on blood vessels and suggested PPARδ may be a new target for hypertension therapy.

## Compliance with Ethics Requirements

*All Institutional and National Guidelines for the care and use of animals (fisheries) were followed.*

## Declaration of Competing Interest

*The authors declare that they have no known competing financial interests or personal relationships that could have appeared to influence the work reported in this paper.*
