# Supplementary material for: Endogenous hydrogen sulfide improves vascular remodeling through PPARδ/SOCS3 signaling
Source: J Adv Res. 2020 Jun 20;27:115–25. doi: 10.1016/j.jare.2020.06.005 (PMC7728593; doi:10.1016/j.jare.2020.06.005)

Supplemental Figure 1. Blood pressure measurement.

A-C: there were lowered CSE protein levels in PPG treated mice and no changes were observed with CBS and MPST expressions. D: PPG treatment were validated by H_2_S measurement. E: PPG group mice exhibited higher mean blood pressure which were modified by NaHS chronic treatment. F: there were no obvious differences in ratio of lumen/outer diameter. Data are means ± SEM. n=6 for each group. ^*^*P*<0.05 versus vehicle, ^#^*P*<0.05 versus PPG.

Supplemental Figure 2. PPG led a time-dependent and dose-dependent reduction of PPARδ and SOCS3 expression, and increase of phosphorylation of STAT3.

A-F: PPG treatment facilitated p-STAT3 expression and downregulated expression of PPARδ, SOCS3 in VSMCs at a time-dependent or dose dependent manner. Data are means ± SEM. n=6 for each group. ^*^*P*<0.05 versus control.

Supplemental Figure 3. The changes of CSE, CBS and MPST with PPG treatment in VSMCs.

A-D: there were lowered CSE protein levels in PPG treated VSMCs and no changes were observed with CBS and MPST expressions in PPG group. Data are means ± SEM. n=6 for each group. ^*^*P*<0.05 versus control.

Supplemental Figure 4. PPG induced collagen production and phenotype transformation of VSMCs.

A: representative images of collagen I, MMP9, αSMA, p27, PCNA, Cyclin E in VSMCs. B-G: expression of collagen I, MMP9, PCNA, Cyclin E were increased, while the production of αSMA, p27 were decreased in PPG treated VSMCs. Data are means ± SEM. n=6 for each group. ^*^*P*<0.05 versus control.

Supplemental Figure 5. PPG promoted inflammatory molecules production and inhibited PPARδ, SOCS3 expression of VSMCs.

A: western blot bands of TNFα, IL6, IL1β, PPARδ, SOCS3, p- STAT3 in PPG treated VSMCs. B-G: quantification results showed increased expression of TNFα, IL6, IL1β and p-STAT3 (Ser 727) and decreased expression of PPARδ, SOCS3 in PPG treated VSMCs. Data are means ± SEM. n=6 for each group. ^*^*P*<0.05 versus control.

Supplemental Figure 6. GW501516 attenuated collagen production and promoted phenotype transformation of VSMCs.

A-F: increased expression of collagen I, MMP9, PCNA, Cyclin E and decreased production of αSMA, p27 were in PPG treated VSMCs could be normalized by GW501516 culture. Data are means ± SEM. n=6 for each group. ^*^*P*<0.05 versus control, ^#^*P*<0.05 versus PPG.

Supplemental Figure 7. PPARδ agonist attenuated inflammatory molecules production in VSMCs.

A-C: increased expression of TNFα, IL6, IL1β in PPG treated VSMCs could be normalized by GW501516 culture. D-F: expressions of CSE, CBS, MPST were not affected by GW501516. Data are means ± SEM. n=6 for each group. ^*^*P*<0.05 versus control, ^#^*P*<0.05 versus PPG.

Supplemental Figure 8. GSK0660 promoted collagen production and phenotype transformation of VSMCs.

A-F: productions of collagen I, MMP9, PCNA, Cyclin E were increased and αSMA, p27 were decreased by GSK0660 culture. Data are means ± SEM. n=6 for each group. ^*^*P*<0.05 versus control.

Supplemental Figure 9. PPARδ antagonist promoted inflammatory molecules production in VSMCs.

A-C: expressions of TNFα, IL6, IL1β in NaHS treated VSMCs could be increased by GSK0660 culture. D-F: expressions of CSE, CBS, MPST were not affected by GSK0660 stimulation. Data are means ± SEM. n=6 for each group. ^*^*P*<0.05 versus control.

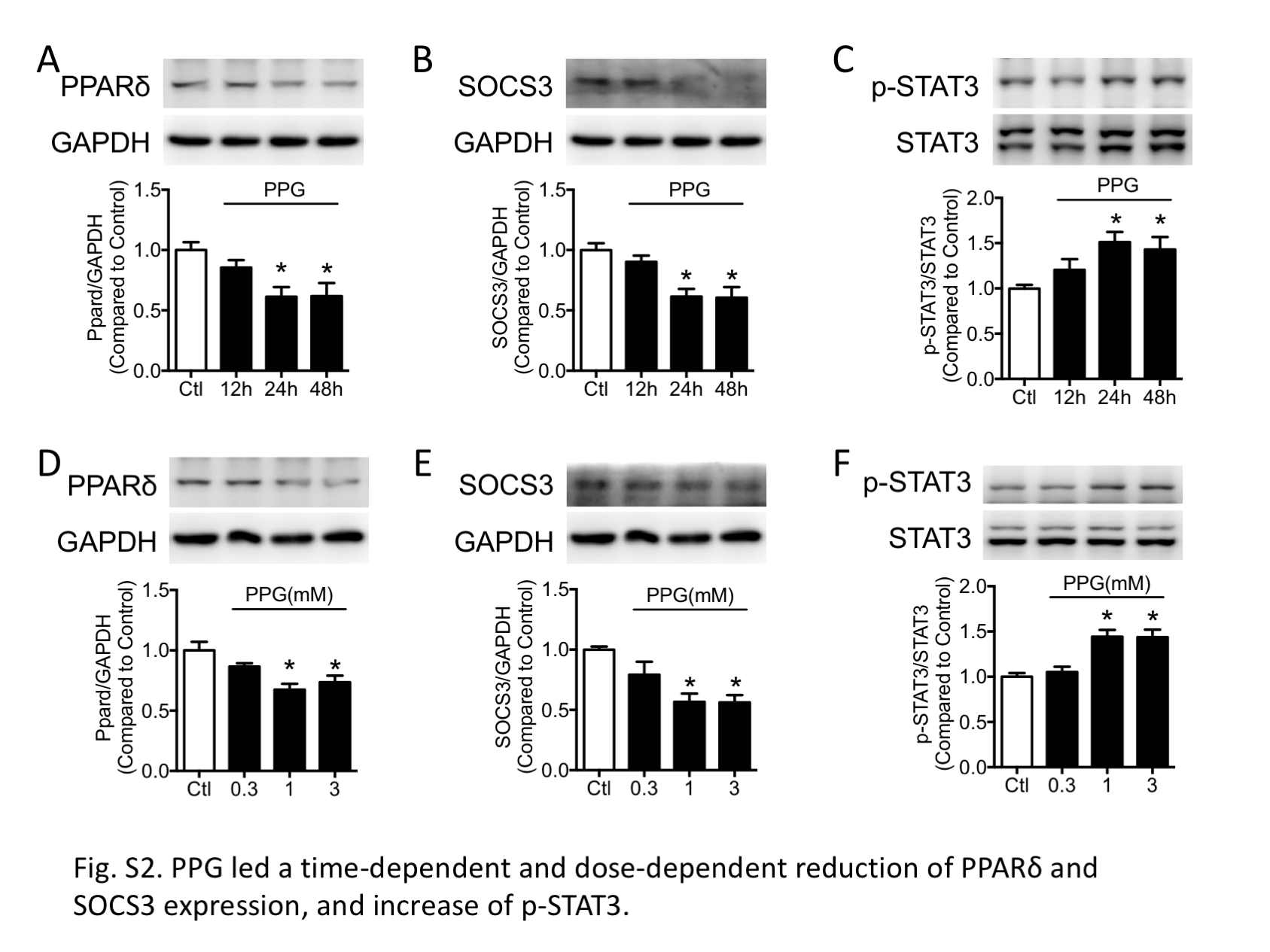


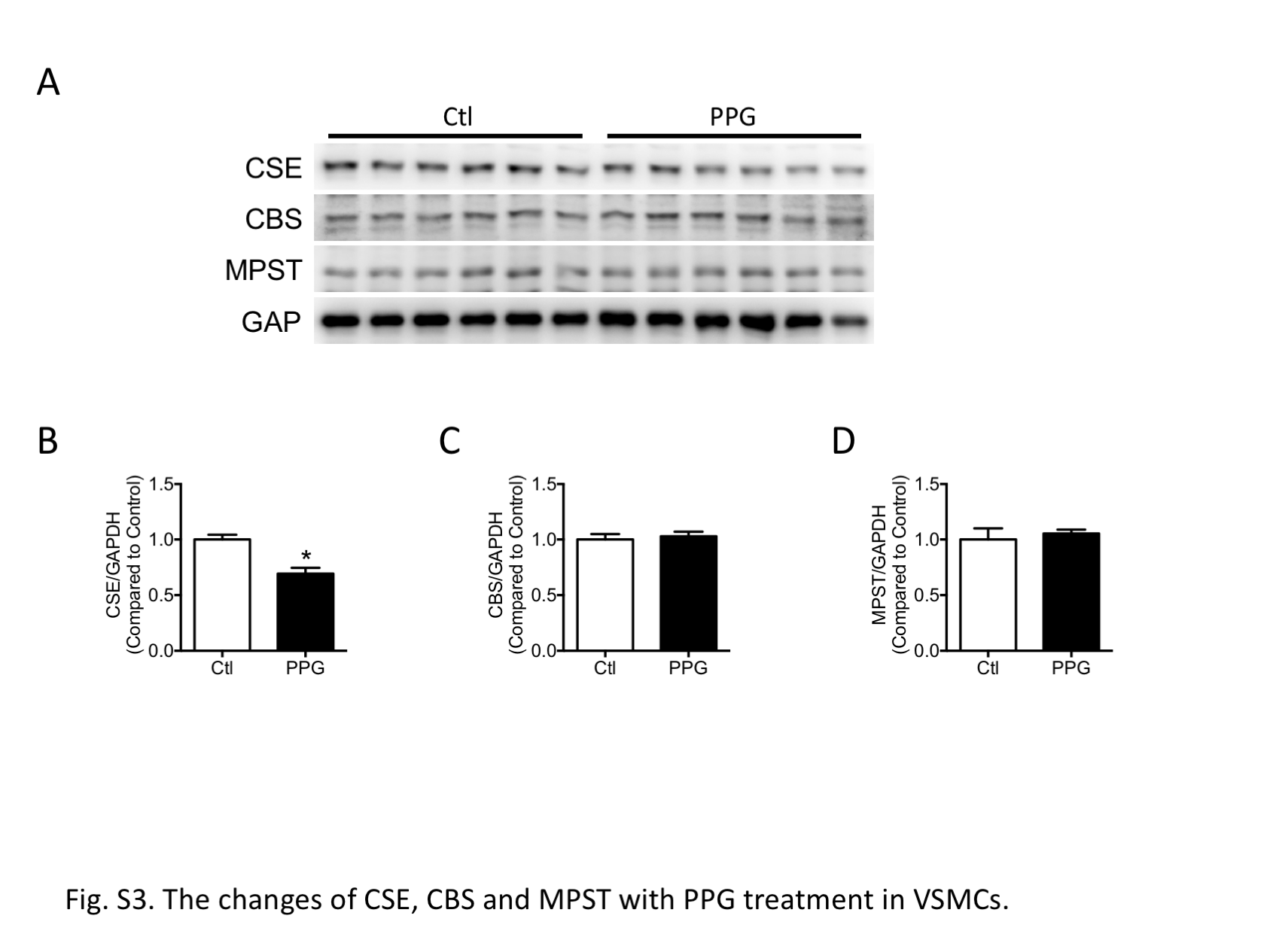


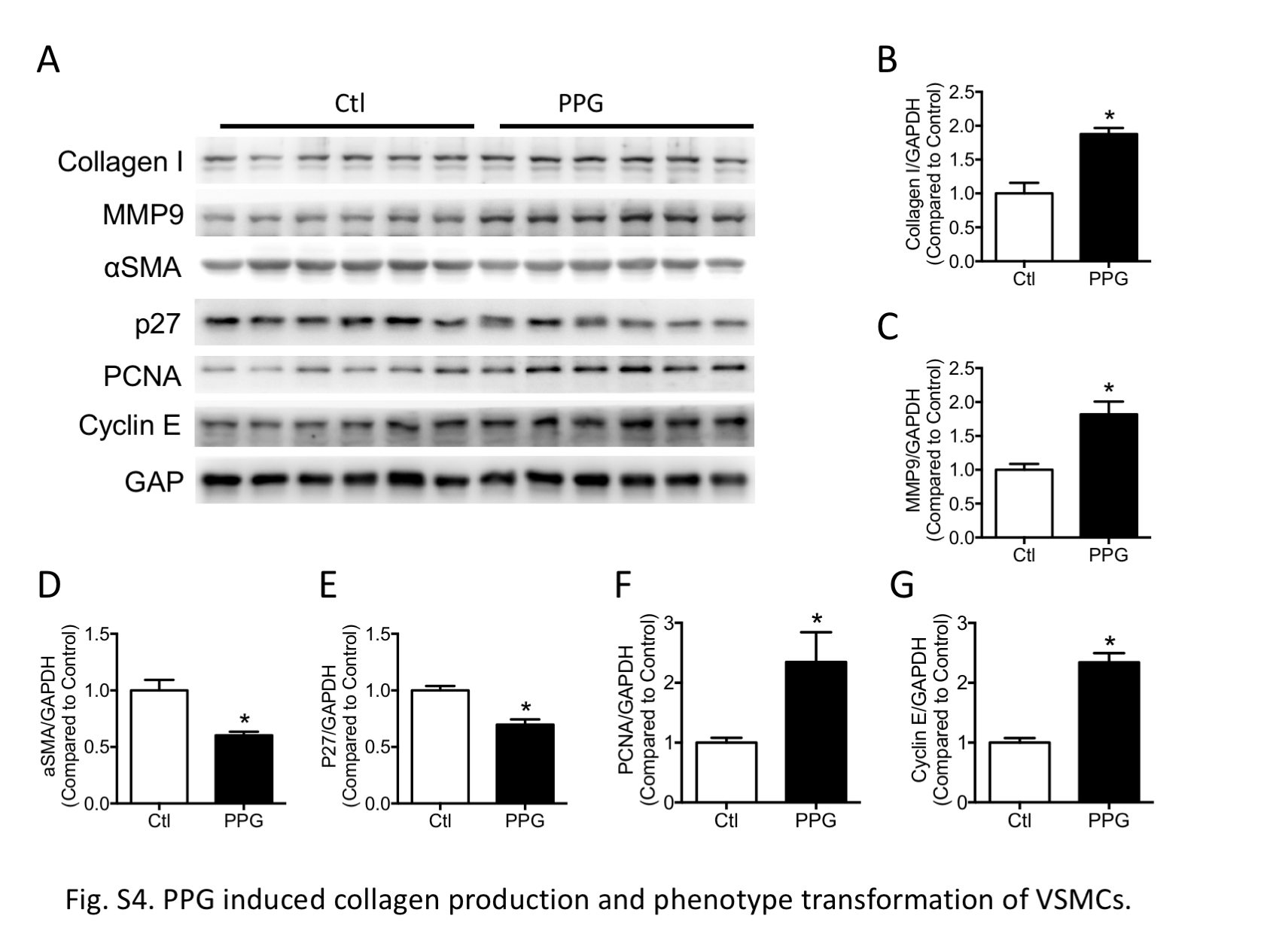


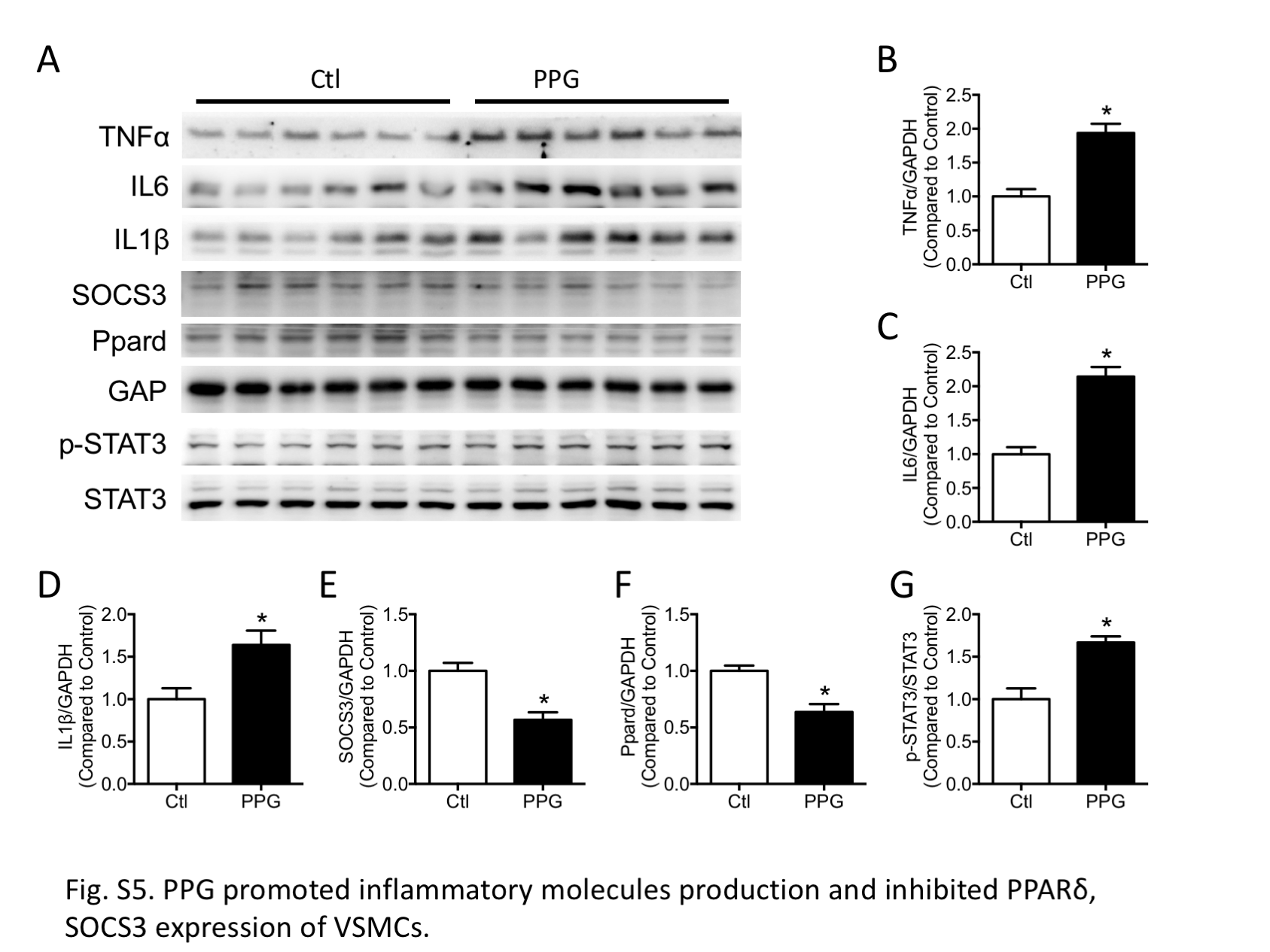

Supplement: Supplementary data 1 [file mmc1.docx]
